# Off-label use of cinacalcet in pediatric primary hyperparathyroidism: A French multicenter experience

**DOI:** 10.3389/fped.2022.926986

**Published:** 2022-08-24

**Authors:** Julie Bernardor, Sacha Flammier, Jean-Pierre Salles, Cyril Amouroux, Mireille Castanet, Anne Lienhardt, Laetitia Martinerie, Ivan Damgov, Agnès Linglart, Justine Bacchetta

**Affiliations:** ^1^Centre de Référence des Maladies Rares du Calcium et du Phosphore, Centre de Référence des Maladies Rénales Rares, Filières de Santé Maladies Rares OSCAR, ORKID et ERKNet, Service de Néphrologie Rhumatologie et Dermatologie Pédiatriques, Hôpital Femme Mère Enfant, Bron, France; ^2^INSERM UMR S1033 Research Unit, Lyon, France; ^3^Service de Néphrologie Pédiatrique, CHU de Nice, Hôpital Archet, Nice, France; ^4^Faculté de Médecine, Université Côte d'Azur, Nice, France; ^5^Centre de Référence des Maladies Rares du Calcium et du Phosphore, Unité d'Endocrinologie, Génétique et Pathologies Osseuses, Filières Santé Maladies Rares OSCAR et BOND, Hôpital des Enfants, Toulouse, France; ^6^Service d'Endrocrinologie et Néphrologie Pédiatrique, Filière de Santé Maladies Rares OSCAR, Hôpital Arnaud de Villeneuve - CHU Montpellier, Université de Montpellier, Montpellier, France; ^7^Centre de Référence des Maladies Rares du Calcium et du Phosphore, Département de Pédiatrie, Filière Santé Maladies Rares OSCAR, CHU Rouen, Rouen, France; ^8^Service de Pédiatrie, CHU de Limoges, Limoges, France; ^9^Service d'Endocrinologie Pédiatrique, Centre de Référence des Maladies Endocriniennes Rares de la Croissance et du Développement (CRMERCD), Hôpital Robert Debré, Assistance Publique - Hôpitaux de Paris, Paris, France; ^10^Center for Pediatric and Adolescent Medicine, University of Heidelberg, Heidelberg, Germany; ^11^Institute of Medical Biometry and Informatics, University of Heidelberg, Heidelberg, Germany; ^12^AP-HP, Centre de référence des maladies rares du métabolisme du calcium et du phosphate, Plateforme d'expertise maladies rares Paris Saclay, filière OSCAR, EndoRare and BOND ERN, Hôpital de Bicêtre Paris Saclay, Le Kremlin-Bicêtre, France; ^13^Université Paris-Saclay, AP-HP, Service d'endocrinologie et diabète de l'enfant, Service de médecine des adolescents, Hôpital de Bicêtre Paris Saclay, INSERM U1185, Le Kremlin-Bicêtre, France; ^14^Faculté de Médecine Lyon Est, Université de Lyon, Lyon, France

**Keywords:** children, primary hyperparathyroidism, cinacalcet, hypercalcemia, Calcium-sensing Receptor (CaSR)

## Abstract

**Background:**

Cinacalcet is a calcimimetic approved in adults with primary hyperparathyroidism (PHPT). Few cases reports described its use in pediatric HPT, with challenges related to the risk of hypocalcemia, increased QT interval and drug interactions. In this study, we report the French experience in this setting.

**Methods:**

We retrospectively analyzed data from 18 pediatric patients from 7 tertiary centers who received cinacalcet for PHPT. The results are presented as median (interquartile range).

**Results:**

At a median age of 10.8 (2.0–14.4) years, 18 patients received cinacalcet for primary HPT (*N* = 13 inactive *CASR* mutation, *N* = 1 *CDC73* mutation, *N* = 1 multiple endocrine neoplasia type 1, N=3 unknown etiology). Cinacalcet was introduced at an estimated glomerular filtration rate (eGFR) of 120 (111–130) mL/min/1.73 m^2^, plasma calcium of 3.04 (2.96–3.14) mmol/L, plasma phosphate of 1.1 (1.0–1.3) mmol/L, age-standardized (z score) phosphate of −3.0 (−3.5;−1.9), total ALP of 212 (164–245) UI/L, 25-OHD of 37 (20–46) ng/L, age-standardized (z score) ALP of −2.4 (−3.7;−1.4), PTH of 75 (59–123) ng/L corresponding to 1.2 (1.0–2.3)-time the upper limit for normal (ULN). The starting daily dose of cinacalcet was 0.7 (0.6–1.0) mg/kg, with a maximum dose of 1.0 (0.9–1.4) mg/kg per day. With a follow-up of 2.2 (1.3–4.3) years on cinacalcet therapy, PTH and calcium significantly decreased to 37 (34–54) ng/L, corresponding to 0.8 (0.5–0.8) ULN (*p* = 0.01), and 2.66 (2.55–2.90) mmol/L (*p* = 0.002), respectively. In contrast, eGFR, 25-OHD, ALP and phosphate and urinary calcium levels remained stable. Nephrocalcinosis was not reported but one patient displayed nephrolithiasis. Cinacalcet was progressively withdrawn in three patients; no side effects were reported.

**Conclusions:**

Cinacalcet in pediatric HPT can control hypercalcemia and PTH without significant side effects.

## Introduction

Pediatric primary hyperparathyroidism (PHPT) is an uncommon endocrine disorder secondary to an increased secretion of parathyroid hormone (PTH), with an incidence of 2–5 per 100 000 children, responsible for hypercalcemia and hypophosphatemia (1). Heterogenous clinical features such as polyuria, nausea, constipation, abdominal pain and failure to thrive, may be the presenting symptoms of chronic hypercalcemia (2). The diagnosis is usually made in children with hypercalcemia with elevated or non-adapted “normal” PTH levels; genetic screening is recommended, especially because of the risk of adenocarcinoma of the parathyroid with specific mutations as in the *CDC73* gene.

In children, single benign parathyroid adenoma may cause PHPT occurring during adolescence and requires parathyroid resection for definitive treatment (1, 3). PHPT may also be part of genetic endocrine syndromes including 1/ Multiple endocrine neoplasia (MEN) syndromes, type I (PHPT, duodenopancreatic neuroendocrine tumors and/or pituitary adenomas, *MEN1* gene mutation) (4), or type II (PHPT, medullary thyroid carcinoma and/or pheochromocytoma, *RET* proto-oncogene mutation) (5); 2/ Hereditary hyperparathyroidism-jaw tumor (HPT-JT) syndrome secondary to *CDC73* mutation (also known as *HRPT2*) (3); and 3/ Inactive Calcium-sensing Receptor (CaSR) secondary to genetic disorders (*AP2S1, GNA11, CASR* mutations) or anti-CaSR antibodies (6, 7). Patients with *CASR* mutations can display parathyroid hyperplasia or adenoma (3).

In 2014, the Fourth international workshop proposed indications for surgery in adults with asymptomatic PHPT more frequently than the last set of guidelines (8). This workshop published surgical management of asymptomatic primary hyperparathyroidism and stated that age below 50 years should be considered as one of indications for surgery (9). Children with sporadic PHPT, symptomatic MEN1 and HPT-JT, should be treated surgically if there are no contraindications (9, 10). In other cases or before surgery, medical management is proposed, aiming at decreasing calcium levels, obtaining normal phosphate levels, repleting native vitamin D, and avoiding hypercalciuria that may further induce nephrolithiasis and/or nephrocalcinosis (8). In adults, the conventional management uses bisphosphonates, with a preference to alendronate, to improve Bone Mass Density (BMD), since it does not alter serum calcium and PTH concentration; and in an acute setting in case of symptomatic severe hypercalcemia, calcimimetics are combinate (8). Restriction of calcium intake below established guidelines for all individuals is not recommended to avoid bone defects and impaired peak bone mass (11).

The calcimimetic cinacalcet inhibits PTH secretion through the sensitization of the parathyroid CaSR by enhancing signal transduction (12). In adults, the recent international guidelines recommend to use calcimimetics in PHPT patients unable to undergo surgical parathyroidectomy (8). A recent meta-analysis of 28 studies, including four randomized clinical trials (RCTs), has reported that serum levels of calcium and PTH levels were significantly reduced, and phosphate levels significantly increased with cinacalcet use in PHPT (13).

Cinacalcet was recently licensed in Europe in dialysis children above 3 years with secondary hyperparathyroidism due to end-stage kidney disease; European guidelines have delineated its use in this peculiar setting (14). Alternatively, the use of cinacalcet, as an off-label therapy in PHPT, has been reported only in few pediatric case reports and is considered to be a challenge in daily practice, because of the putative risk of hypocalcemia, increased QT interval and drug interactions (15, 16).

Thus, we aimed to retrospectively report the French experience of cinacalcet in pediatric PHPT. We hypothesized that cinacalcet improves calcium levels with a good safety profile.

## Patients and methods

### Patients

This is a retrospective multicenter series of 18 pediatric patients (below the age of 18 years), followed in seven tertiary centers of rare diseases of calcium and phosphate metabolism (Toulouse *N* = 6; Montpellier *N* = 4; Lyon *N* = 2; Bicêtre Paris Saclay *N* = 2; Rouen *N* = 2; Paris-Robert Debré *N* = 1; Limoges *N* = 1), in France, for PHPT. We included all children who started cinacalcet between November 2013 and January 2020, as an off-label therapy, with at least 1 year of follow-up for 17 patients.

### Biochemicals and assessment of growth parameters

As part of the routine follow-up of these patients with PHPT, total calcium, phosphate, magnesium, alkaline phosphatase (ALP), PTH, creatinine, 25-OH vitamin D (25-OHD) and urinary calcium were regularly assessed. Calcium (Ca), phosphate (Ph), creatinine, and 25-OHD levels were locally measured by standard methods. Variables with age-dependent reference values (plasma phosphate concentration, ALP, urinary calcium/creatinine ratio) are expressed as z score, calculated as follows: z score = (measured value – mean normal value)/SD. Reference values for across corresponding age groups and/or gender were used from prior literature (17, 18) whereby a square root transformation and corresponding reference values for ALP (19). Estimated Glomerular Filtration Rate (eGFR) was estimated using the 2009 Schwartz formula (20). Laboratory parameters were collected at six different time points: initiation of cinacalcet therapy (baseline), and after 1, 3, 6, and 12 months; data at last follow-up were also recorded. In the youngest children in whom 24-h urinary calcium assessment was not possible, spot mictions were obtained to measure urinary calcium/creatinine ratio.

Because the local assays used for routine assessment of PTH were different, as illustrated in Table 1, we decided to present PTH data as xx-time the upper limit for normal (xx-ULN).

**Table 1:** Assays used for routine assessment of plasma PTH levels in different contributing centers.

**Commercial kit**	**Center**	**Assay**	**Normal range (ng/L)**
Roche on a Cobas analyzer	Lyon, Montpellier, Toulouse	Electro-CLIA	15–65
Liaison 1-84 PTH Assay, DiaSorin	Limoges	CLIA	6.5–36.8
Liaison XL Assay, DiaSorin	Rouen	CLIA	5–40
Access Intact, Beckman Coulter	Paris-Robert Debré	CLIA	10–50
Centaur, Siemens, Deerfield	Paris-Bicêtre	CLIA	17–84

CLIA, chemi-luminiscence-immuno assay.

Main outcome parameters [height, weight and Body Mass Index (BMI)] were referenced to sex and age according to new French growth curves (www.afpa.org) and are reported as standard deviation scores (SDS).

### Ethics

The study was approved by the local ethical committee (*Comité d'Ethique des Hospices Civils de Lyon*, session 18/02/2019, approval 19-26), and declared to the Information Technology and Liberty Commission (CNIL n°19-168). As per French law, parents and patients do not need to give a written consent for retrospective studies.

### Statistical analysis

Non-parametric Kruskal-Wallis tests followed by Dunn's tests compared to baseline were used. In all cases, *p*-values below 0.05 were considered statistically significant using GraphPad Prism software 8.0 (GraphPad, La Jolla, CA, USA). Results are presented as median (inter-quartile range, i.e., 25th−75th percentile) for biochemical routine parameters.

## Results

### Cinacalcet introduction

Relevant demographic, clinical and biochemical features of the 18 patients (8 girls) at the time of cinacalcet initiation are presented in Table 2. At a median age of 10.8 (2.0–14.4) years, cinacalcet was introduced at an estimated glomerular filtration rate (eGFR) of 120 (111–130) mL/min/1.73 m^2^, plasma calcium of 3.04 (2.96–3.14) mmol/L, plasma phosphate of 1.1 (1.0–1.3) mmol/L, age-standardized (z score) phosphate of −3.0 (−3.5;−1.9), total ALP of 212 (164–245) UI/L age-standardized (z score) ALP of −2.4 (−3.7;−1.4), 25-OHD of 37 (20-46) ng/L, and PTH of 75 (59–123) ng/L corresponding to 1.2 (1.0–2.3)-time the ULN. The starting daily dose of cinacalcet was 0.7 (0.6–1.0) mg/kg, with a maximum dose of 1.0 (0.9–1.4) mg/kg per day (median in CaSR group: 1.1 (0.9–1.3) mg/kg/day; other etiologies: 1.7 (1.3–1.9) mg/kg/day). Six patients (32%) had received bisphosphonates (Pamidronate disodium) and no parathyroidectomy was performed before cinacalcet introduction. Seventeen patients had at least a 1-year follow-up. The median time of follow-up was 2.2 (1.3–4.3) years. No patients have received phosphate supplementation and active vitamin D analogs during the follow-up. Main outcome parameters (height, weight and BMI) remained stable during the follow-up (Table 3). Nephrocalcinosis was improved for one patient and another one displayed nephrolithiasis secondary to hypercalciuria (this patient had nephrolithiasis history before the Cinacalcet introduction).

**Table 2:** Patient characteristics at Cinacalcet initiation and during follow-up.

**No**.	**Etiology**	**Age at Cina intro (years)**	**Sex**	**SDS height**	**SDS weight**	**BMI (kg/m^2^)**	**PTH (ng/L)**	**xx-ULN PTH**	**Ca (mmol/L)**	**SDS Ph**	**Biphosphonates (Pamidronate disodium), before Cinacalcet, dose (mg/kg); number of doses**	**Cinacalcet initiation (mg/kg/day)**	**Cinacalcet max dose (mg/kg/day)**	**Cinacalcet dose at last follow-up (μg/kg/day)**
1	NSHPT Ho *CASR* mutation c.413C>T ; p.Thr138Met	0.1	F	0	0	14.7	124	1.9	2.98	−3.9	No	1.0	1.0	0.6
2	NSHPT Ho *CASR* mutation c.1972C>T ; p.Arg648Ter	0.1	F	−2	−0.5	14.3	459	7.1	3.04	−4.1	0.5; 2	0.1	0.7	0.6
3	NSHPT Ho *CASR* mutation c.1745G>A; p. Cys582Tyr	0.2	M	1	−0.5	13.9	34	0.9	3.16	−4.3	NA; 2	0.4	0.9	0.1
4	FHH He *CASR* mutation c.2425T>C ;p.Phe809Leu	1.1	M	1	−1	14.8	53	0.8	3.12	−3.1	No	1.6	3.2	1.8
5	FHH He *CASR* mutation c.1972C>T ; p.Arg648Ter	1.4	F	0	0.5	17.1	14	0.2	2.88	−1.6	1; 5	0.4	1.4	0.2
6	FHH He *CASR* mutation c.2425T>C ;p.Phe809Leu	3.6	M	0.5	1	16.4	80	1.2	3.07	−3.6	NA; 1	0.9	1.4	1.3
7	FHH He *CASR* mutation c.2425T>C ;p.Phe809Leu	6.6	F	1	2	22.1	59	0.9	2.96	−3.2	No	0.7	1.3	1.3
8	FHH He *CASR* mutation c.2425T>C ;p.Phe809Leu	7.3	M	−1.5	−0.5	15.5	67	1	2.86	−2.9	No	1.2	1.5	1.5
9	FHH He *CASR* mutation c.1745G>A; p. Cys582Tyr	10.1	F	1	2	24.2	85	1.3	3.10	−1.2	No	1.1	1.1	1.1
10	FHH He *CASR* mutation c.2425T>C ; p.Phe809Leu	11.5	F	1	1	21.8	60	0.9	3.23	−1.5	No	0.6	0.6	NA
11	FHH He *CASR* mutation c.413C>T ; p.Thr138Met	12.2	M	0	−1	16.9	72	1.1	2.80	−1.8	No	0.9	0.9	NA
12	FHH Ho *CASR* mutation c.482A>G ; p.Tyr161Cys	12.6	M	−0.5	0	19.1	59	1.2	2.99	−2.5	No	0.7	1.0	0.9
13	FHH Ho *CASR* mutation c.482A>G ; p.Tyr161Cys	14.7	F	−0.5	0	20.5	243	4.9	3.40	−3.6	No	0.6	1.2	1.2
14	Hereditary hyperparathyroidism-jaw tumor syndrome *CDC73* mutation	14.3	F	−1	−0.5	20.0	171	2.6	2.86	−2.8	No	0.6	1.9	1.8
15	MEN type 1 *MEN1* He mutation	14.4	M	−1.5	−2	16.9	77	1.2	2.87	−1.5	No	0.8	1.3	1.2
16	Unknown	15.3	M	NA	−2	15.3	199	2.4	3.33	−3.3	NA; 2	1.1	1.9	2.1
17	Unknown	15.5	M	0.5	−1	23.0	38	1	3.04	−3.0	No	1.7	1.7	NA
18	Unknown	17.5	M	1	0.5	24.9	120	3	2.96	−2.0	No	0.7	0.7	NA

N°., number; NSHPT, Neonatal Severe Hyperparathyroidism; FHH, Familial Hypocalciuria hypercalcemia; He, heterozygous; CASR, Calcium sensing receptor; MEN, Multiple Endocrine Neoplasia; Cina, Cinacalcet; Ca, Calcium; SDS, Standard Deviation; NA, not available; Intro, Introduction; ULN, upper limit for normal; SDS Ph, phosphate levels were normalized and expressed as Z-score for age.

**Table 3:** Summary of the biochemical data available in patients at the different follow-up.

	**Baseline**		**1 Month**		**3 Months**		**6 Months**		**12 Months**		**Last follow-up**	
Dose mg/kg/j		0.7 (0.6–1.0)		1.2 (0.6–1.4)		0.8 (0.6–0.9)		1.1 (0.8–1.4)		1.1 (0.8–1.3)		1.3 (0.9–1.7)	
**CaSR**	Others	0.7	0.8	1.0	1.4	0.9	0.7	1.0	1.6	1.0	1.8	1.2	1.7
PTH, g/L		75 (59–123)		59 (46–82)		46 (27–100)		53 (46–98)		41^*^ (30–63)		37^*^ (34–54)	
**CaSR**	Others	67	120	51	159	46	110	48	98	38	65	37	87
xx-ULN PTH		1.2 (1.0–2.3)		0.9 (0.7–1.2)		0.7 (0.5–1.5)		1.0 (0.8–1.4)		0.8^*^ (0.5–1.1)		0.8^*^ (0.5–0.8)	
**CaSR**	Others	1.1	1.9	0.8	3.0	0.7	1.7	0.8	1.5	0.7^*^	1.7	0.7	1.0
Calcium mmol/L		3.04 (2.96–3.14)		2.80^*^ (2.60–2.93)		2.90 (2.79–2.93)		2.67^***^ (2.55–2.80)		2.80^**^ (2.70–2.84)		2.66^**^ (2.55–2.90)	
**CaSR**	Others	3.04	2.96	2.83^*^	2.25^*^	2.91	2.49	2.76^**^	2.56	2.81^*^	2.71	2.85^*^	2.61
Ph mmol/L		1.1 (1.0–1.3)		1.4 (0.9–1.6)		1.2 (1.1–1.5)		1.3 (1.1–1.4)		1.2 (1.1–1.4)		1.2 (1.1–1.2)	
**CaSR**	Others	1.2	0.9	1.4	1.4	1.3	1.2	1.3	0.9	1.2	0.8	1.2	1.1
Ph Z-score		−3.0 (−3.6 to −1.9)		−2.0 (−3.4 to−1.4)		−2.1 (−3.4 to −1.7)		−1.9 (−2.6 to −1.6)		−2.3 (−2.9 to −1.8)		−2.2 (−2.6 to −1.4)	
**CaSR**	Others	−3.1	−2.9	−2.0	−1.8	−2.7	−1.5	−1.9	−2.8	−2.2	−3.1	−2.4	−1.7
eGFR mL/min/1.73 m^2^		120 (111–130)		130 (114–136)		121 (115–125)		109 (96–123)		110 (100–130)		114 (95–121)	
**CaSR**	Others	127	112	136	119	120	NA	109	105	103	137	114	96
25-OHD ng/L		37 (20–46)		35 (29–38)		25 (21–36)		34 (24–45)		35 (24–45)		33 (21–41)	
**CaSR**	Others	42	18	35	NA	25	NA	38	26	35	29	33	30
ALP UI/L		212 (164–245)		253 (220–287)		304 (277–369)		185 (139–240)		254 (174–305)		170 (158–196)	
**CaSR**	Others	220	217	253	NA	304	NA	204	130	295	139	170	NA
ALP Z-score		−2.4 (−3.7 to −1.4)		−2.8 (−3.1 to −2.6)		−1.9 (−2.6 to 0)		−2.6 (−3.4 to −1.8)		−1.5 (−2.7 to −0.9)		−3.0 (−3.3 to −2.3)	
CaU mmol/L		0.6 (0.1–1.6)		0.9 (0.1–7.8)		0.2 (0.2–0.6)		2.0 (1.0–7.1)		0.7 (0.3–4.1)		0.6 (0.5–2.3)	
**CaSR**	Others	0.1	6.5	0.3	6.5	0.2	NA	1.1	6.0	0.4	5.6	0.6	2.2
Ca/CreatU mmol/mmol		0.1 (0.0–0.6)		0.2 (0.1–0.8)		0.1 (0.1–0.1)		0.3 (0.1–0.4)		0.2 (0.1–0.4)		0.2 (0.1–0.2)	
**CaSR**	Others	0.0	0.3	0.1	0.2	0.1	NA	0.2	0.4	0.2	0.2	0.2	0.2
Ca/CreatU Z-score		−0.8 (−1.0 to 0.3)		−0.6 (−0.9 to 2.9)		−0.9 (−0.9 to −0.9)		−0.6 (−0.9 to −0.6)		−0.4 (−0.7 to 0.4)		−0.3 (−0.8 to −0.3)	
**CaSR**	Others	−0.9	0.9	−0.6	−0.2	−0.9	NA	−0.6	−0.9	−0.5	−0.3	−0.6	−0.3
SDS height		0 (−0.5 to 1)		0 (−0.5 to 1)		−0.5 (−1 to 0)		−0.5 (−1 to 0.5)		0 (−1 to 0.5)		0 (−1 to 1)	
**CaSR**	Others	0	0.5	0	−0.5	0	−1	−0.5	−1	0	−1	0	−1
SDS weight		0 (−1 to 0.5)		−0.5 (−1 to 1)		0 (−0.5 to 0)		0 (−0.5 to 0.5)		−0.5 (−1 to 0)		0 (−0.5 to 1.5)	
**CaSR**	Others	0	−1	0.5	−1	0	−1	0	−0.5	0	−0.5	−0.5	0

CaSR, Calcium Sensing receptor patients; ULN, upper limit for normal; eGFR, Estimated Glomerular Filtration Rate; Ph Z-score, phosphate Z-score; 25-OHD, 25-OH vitamin D; CaU, urinary calcium; CreatU, creatininuria; ALP, total Alkaline Phosphatase; NA, not available; SDS, Standard Deviation. The results are presented as median (interquartile range) and median is presented for CASR mutation patients and patients with other etiologies.

Gray cellules were different statistical data between baseline and different follow-ups after this initiation of Cinacalcet for biological results. Statistical analyses were performed with Kruskall–Wallis test

* p < 0.05,

** p < 0.01

and

*** p < 0.001.

### Evolution of PTH and calcium levels in response to cinacalcet

**Figure 1:**
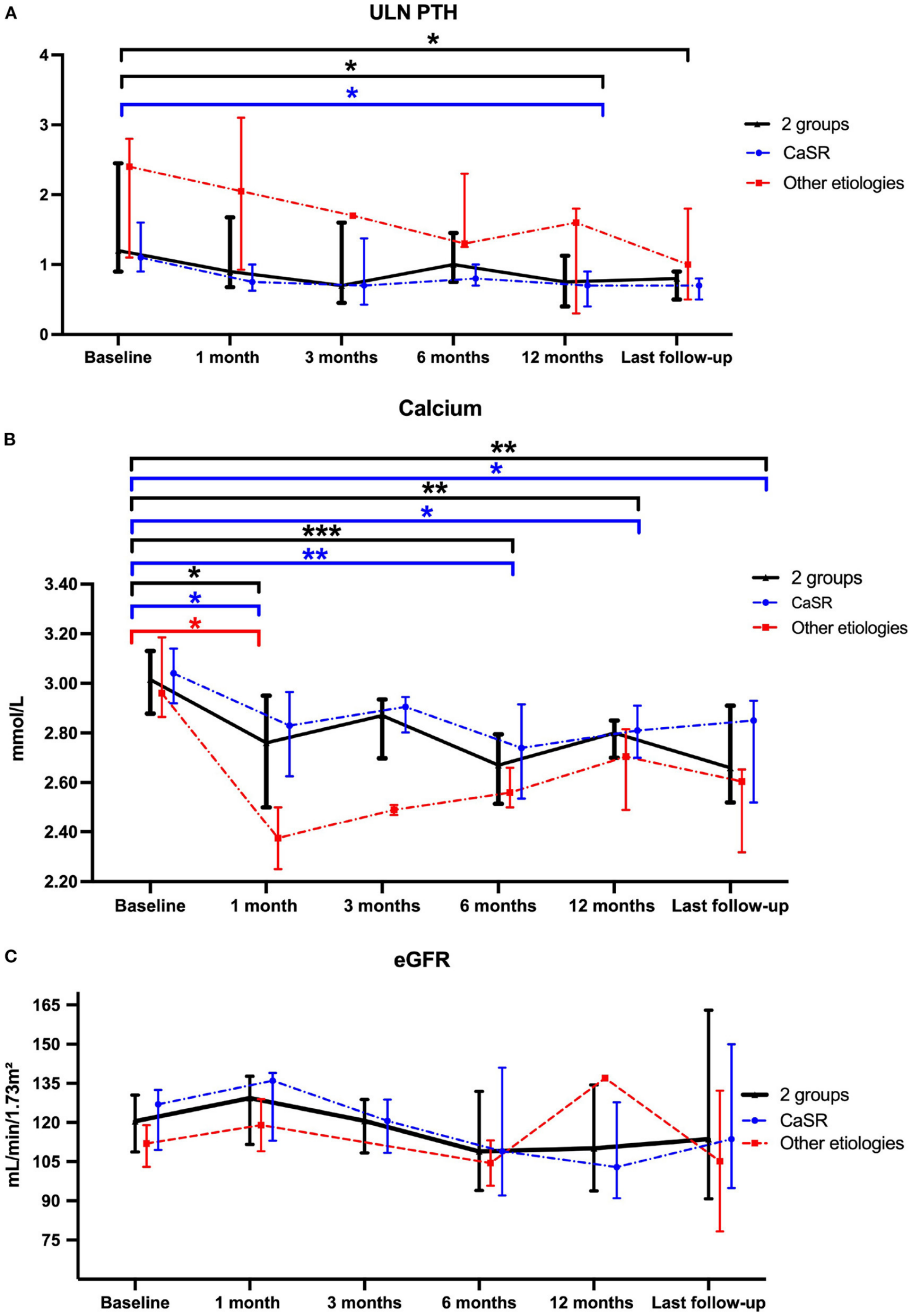
Comparison of ULN-PTH **(A)**, total calcium **(B)** and eGFR **(C)** data at Cinacalcet cinacalcet initiation and during follow-up. Each dot on the graph represents median with interquartile range of different biological data levels at the different time-points. Statistical analyses were performed with Kruskall–Wallis test: *p < 0.05, **p < 0.01 and ***p < 0.001. The blue line represents the “*CASR* mutation patients” sub-group, the red line represents the sub-group of patients without *CASR* mutation and the black line represents these 2 sub-groups.

The evolution of the main biochemical parameters at different time points under cinacalcet therapy is reported in Table 3. We observed a nearly 50% significant decrease in PTH levels between cinacalcet initiation [75 (59–123) ng/L] and the last follow-up [37 (34-54) ng/L]. The ULN PTH levels significantly decreased between cinacalcet initiation and after 12 months (*p* = 0.02), and at the last follow-up (*p* = 0.01), as shown in Figure 1A. We observed a significant decrease in calcium levels at 1, 6, 12 months and at the last follow-up as compared to baseline, with a similar pattern in the CaSR sub-group (Figure 1B). At the time of cinacalcet initiation, all patients were hypercalcemic ≥2.80 mmol/L; calcium levels remained above 2.8 mmol/L in 6 patients after initiation of cinacalcet therapy.

### Evolution of eGFR and urinary calcium in response to cinacalcet

**Figure 2:**
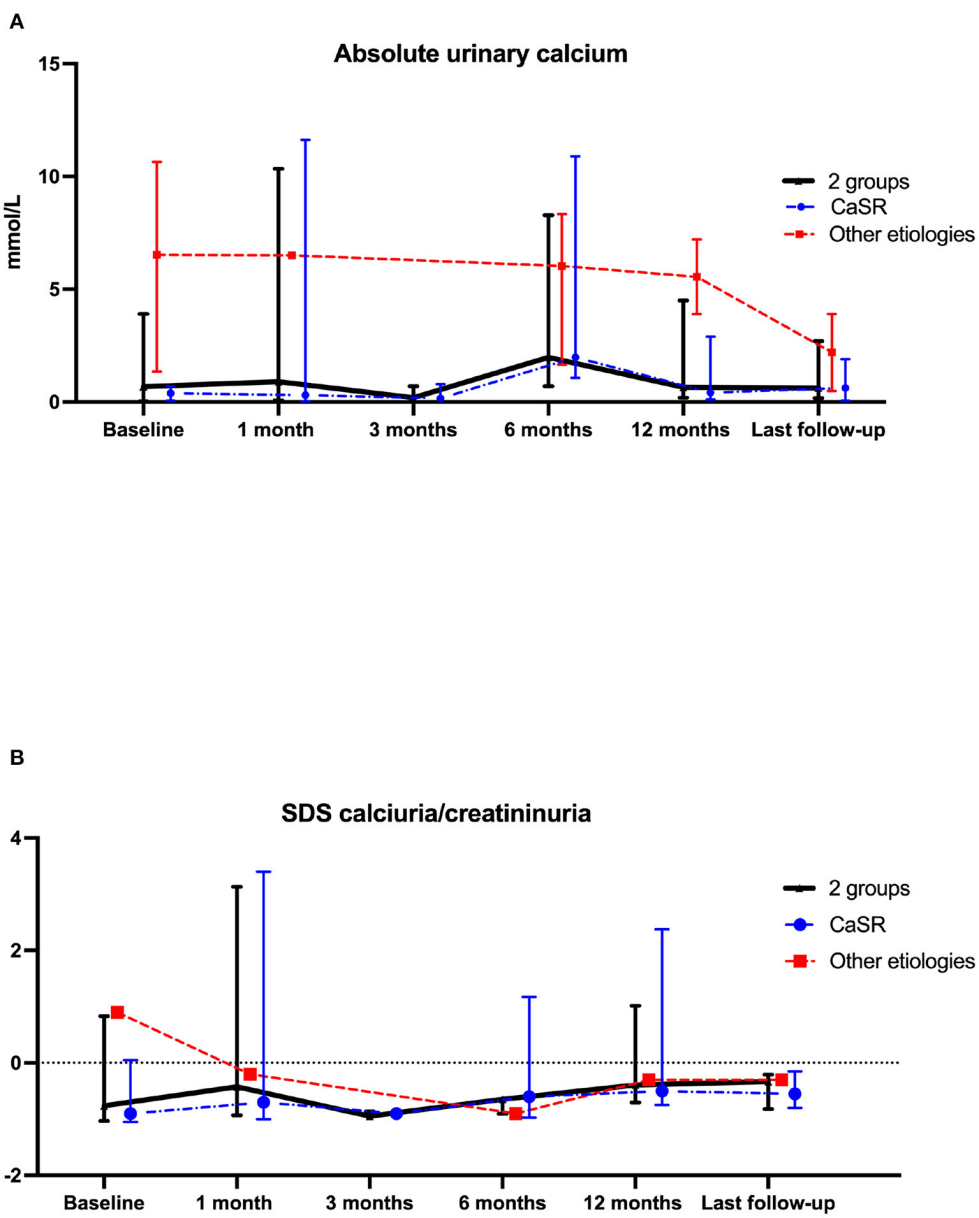
Comparison of calciuria **(A)** and z-score for age calciuria/creatininuria on one urine sample **(B)** data at cinacalcet initiation and during follow-up. Each dot on the graph represents median with interquartile range of biological data at the different time-points. Statistical analyses were performed with Kruskall–Wallis test: *p* = not statistically significant (NS). The blue line represents the “*CASR* mutation patients” sub-group, the red line represents the sub-group of patients without *CASR* mutation and the black line represents these 2 sub-groups.

Renal function assessed by eGFR remained stable during follow-up (Figure 1C). Results were similar in patients with *CASR* mutations as compared to patients with other etiologies (Figure 1C). Urinary absolute calcium excretion and urinary calcium/creatinine ratio remained stable, whatever the underlying cause; as expected patients with *CASR* mutations displayed lower urinary calcium levels (Figure 2). Interestingly, one may discuss a trend toward decreased absolute urinary calcium levels in patients without *CASR* mutations at the end of the follow-up (Figure 2A); this could be relevant in practice since median urinary calcium at baseline in this sub-group is far higher (i.e., 6.5 mmol.L) than the crystallization threshold (i.e., 3.8 mmol/L).

### Evolution of phosphate, ALP, and 25-OHD in response to cinacalcet

**Figure 3:**
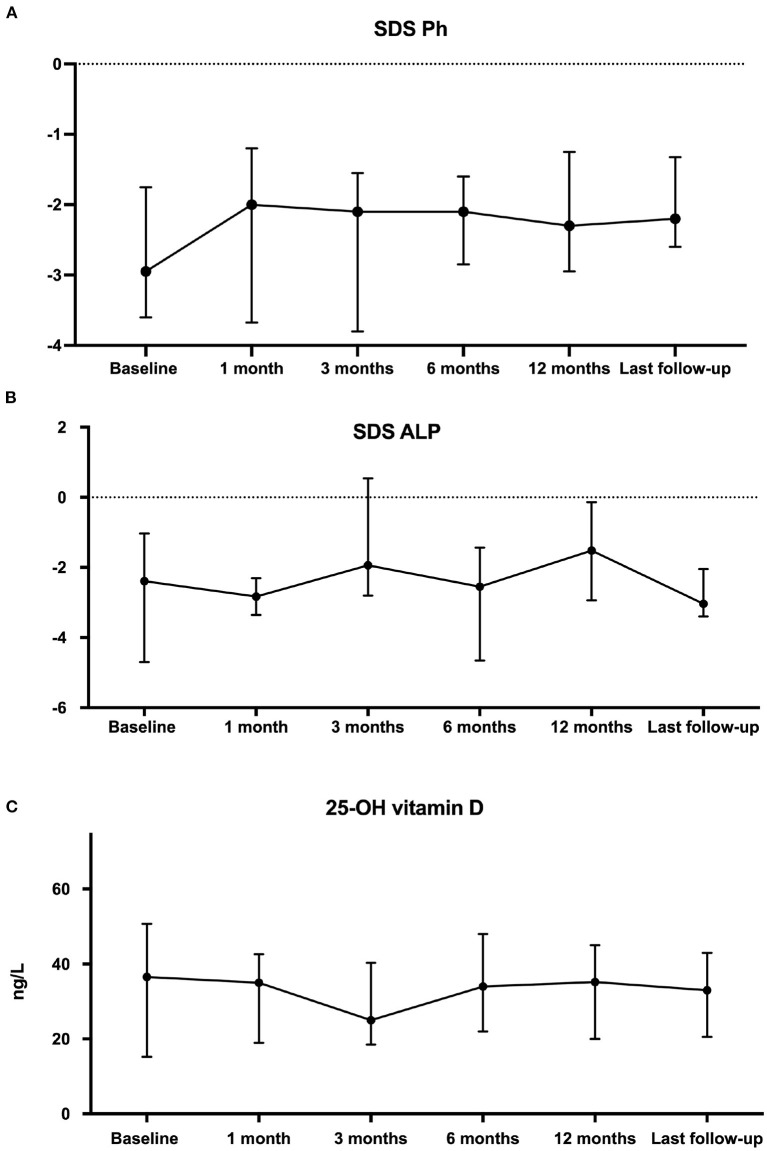
Comparison of phosphate levels as z-score for age **(A)**, z-score for age alkaline phosphatases **(B)** and 25-OH vitamin D **(C)** data at cinacalcet initiation and during follow-up. Each dot on the graph represents median with interquartile range of biological data at the different time-points. Statistical analyses were performed with Kruskall–Wallis test: *p* = not statistically significant (NS).

As shown in Figure 3A, there has been no significant change of phosphate SDS levels before and after cinacalcet therapy. As illustrated in Figure 3B, age-standardized (z score) ALP remained stable. Of note, following European recommendations guidelines (8), 12 children received native vitamin D supplementation to obtain normal 25-OHD levels between 20 and 40 ng/ and 25-OHD levels remained stable during the observational period (Figure 3C).

### Side effects

No severe side effects were reported in 18 patients, and notably no iatrogenic hypocalcemia and no increased QT interval. Renal ultrasounds (when available, *N* = 6 at baseline; *N* = 5 at 1 year and *N* = 6 at the last follow-up) were performed during the follow-up. We observed a disappearance in the last ultrasounds for one patient who presented nephrocalcinosis at baseline. Cinacalcet was withdrawn in three patients: in one patient because of complete calcium normalization after 1 year of follow-up, in one patient because of secondary surgery of an adenoma, and for the last patient because of bad therapeutic compliance.

## Discussion

This retrospective multicenter observational study evaluated the off-label use of cinacalcet to treat pediatric PHPT. The main results are the following: 1/ cinacalcet allows better control of calcium and PTH levels, without any substantial changes in urinary calcium excretion, and 2/ cinacalcet is safe and effective without notable side effects.

In pediatrics, data are scarce, and to our knowledge, only 50 pediatric patients (including the 18 patients presented here) out of a total of almost 1,200 pediatric patients reported in the literature with PHPT, have been treated with cinacalcet, as summarized in Table 4 (15, 16, 21–25, 27–30, 32, 33, 35–37, 40, 43–45). The present study almost doubles the number of reported pediatric patients having received cinacalcet as an off-label drug for PHPT.

**Table 4:** summary of the pediatric studies reporting the use of cinacalcet in children.

**References**	**No. cases**	**Etiology**	***CASR*** **mutation protein**	**Age at inclusion**	**Follow-up**	**Cinacalcet dose**	**Main conclusions: calcium/** **phosphate homeostasis**	**Main conclusions: calciuria/** **nephrocalcinosis**	**Main conclusions: bone turnover**	**Side effects**
Henrich et al. (21)	1	Not available	Not available	16 years	8 weeks	30 mg/day during 4 weeks; then 60 mg/day during 4 weeks	Normalization of calcium; PTH changes were assay dependent	Not evaluated	Not evaluated	No side effects except for mild headache
Alon (22)	1	He *CASR* mutation	c.659G>A p.Arg220Gln	6 years	12 months	30 mg/day during 2 weeks; then 60 mg/day	PTH and ionized Ca decreased; near-normal levels	Not evaluated	Not evaluated	No
Reh et al. (23)	1	He *CASR* mutation	c.554G>A p.Arg185Gln	23 days	31 months	20 mg/m^2^/day	Serum Ca: near-normal levels; PTH decreased	Urine calcium remained undetectable	ALP slightly above normal; at 17 months, osteopenia greatly decreased	No
Wilhelm-Bals et al. (24)	1	Ho *CASR* mutation	c.206G>A p.Arg69His	6 years	5 years	1.4 mg/kg/day increased to 3.5 mg/kg/day and 1.4 mg/kg/day on alternating days	Normalization of calcium, PTH and phosphate levels	Urine calcium levels decreased at the normal range	Osteocalcin and ALP: normal values	No
García Soblechero et al. (25)	1	Ho *CASR* mutation	c.1392_1404del13 p.Arg465Leufs*9	2 months	49 days	20 mg/m^2^/day	Calcemia was maintained at acceptable levels; no significant decrease in the PTH levels	Not evaluated	Not evaluated	No
Atay et al. (26)	1	Ho *CASR* mutation	c222_226delGATAT	21 days	14 months	30 mg/m^2^/d up to 90 mg/m^2/^d	Persistant hyperPTH	Not evaluated	Hungry bone	No
Gannon et al. (27)	1	He *CASR* mutation	c.554G>A p.Arg185Gln	2 days	18 months	6–202 mg/m^2^/day	Reductions in serum concentrations of ionized calcium and intact PTH over the next 4 days	Fractional excretion of calcium remained low	Improvement in bone mineralization and no further fractures	No
Tenhola et al. (28)	1	22q11.2 deletion syndrome and *AP2S1* mutataion	Not applicable	10 years	3 years	30 mg/day to 30 mg twice daily	Plasma calcium levels normalized, and PTH levels decreased slightly	Fractional excretion of calcium remained low	BMD remained stable during the follow-up	Yes: occasional numbness and tingling in his legs
Fisher et al. (29)	2	*He CASR* mutation	c.554G>A p.Arg185Gln	Case 1: 13 months Case 2: 4 months	Case 1: 32 months Case 2: Not available	Case 1: 3.7 mg/kg/day Case 2: 2 mg/kg/day	Cases 1 and 2: Normalization of calcium and PTH levels	Not evaluated	Not evaluated	Case 1: mild nausea and vomiting after one dose increase Case 2: mild nephrocalcinosis
Srivastava et al. (30)	1	Pseudo hypoPTH type 1b	Not applicable	4.8 years	32 months	0.8 mg/kg/day	Serum calcium, phosphorus, PTH and ALP concentrations improved	Kidney function and urine calcium level remained normal	Bone turnover markers and radiographic abnormalities improved under Cinacalcet	No
Savas-Erdeve et al. (31)	1	Ho *CAS*R mutation	c.1630C>T p.Arg544*	12 days	18 months	10 mg to 25 mg/kg/m^2^	Persistant hypercalcemia and hyperPTH	Not evaluated	Not evaluated	No
Murphy et al. (32)	1	Ho *CAS*R mutation	c.206G>A p.Arg69His	4 days	46 days	0.4 mg to 8.5 mg/kg/day	Persistant hypercalcemia and hyperPTH	Not evaluated	Not evaluated	No
Alon et al. (33)	1	XLH Rickets	Not applicable	5 years	18 months	1 mg/kg/day	Normalization of calcium, PTH and phosphate levels	No nephrocalcinosis	BMD remained stable during the follow-up	No
Ahmad et al. (34)	1	Ho *CAS*R mutation	C.1378-2A>G	60 days	16 months	4 mg/kg/day	Persistant hypercalcemia and hyperPTH	Not evaluated	Not evaluated	No
Mogas et al. (35)	2	He *CASR* mutation	Case 2: c.2446A>G+ p.Ile816Val	13 years	Case 1: 18 months Case 2: 12 months	Case 1: 1.1 mg/kg/day Case 2: 30 mg/day	Normalization of calcium and PTH levels	Urine calcium levels decreased at the normal range	Not evaluated	No
Sun et al. (16)	1	Ho *CASR* mutation	c.242T>A p. Ile81Lys	72 days	10 months	30–45 mg/m^2^/day	Total calcium was maintained within the high-normal range and PTH was normalized	Not evaluated	ALP: normal values	No
Capozza et al. (36)	1	Ho *CASR* mutation	IVS5+1G>A c.1608+1G>A Splice site-skipped Exon 5	18 days	10 days	0.4 mg/kg/day	Normalization of calcium and PTH	Not evaluated	Not evaluated	No
Scheers et al. (37)	1	*SPINK1/AP2S1* mutation	Not applicable	13 years	6 years	40 mg/day	Normalization of calcium and PTH	Urine calcium levels remained stable at the normal range	Not evaluated	No
Hashim et al. (38)	1	Ho CASR mutation	c.679 C>T p. Arg227Ter	10 days	18 months	0.5 mg/kg/d up to 11 mg/kg/d	Persistant hypercalcemia and hyperPTH	Not evaluated	Not evaluated	No
Forman et al. (39)	1	He *CASR* mutation	c.554G>A p.Arg185Gln	3 days	14 months	0.4–5 mg/kg/day	Normalization of calcium PTH, and phosphate	No nephrocalcinosis	Not evaluated	No
Sadacharan et al. (40)	4	Ho *CASR* mutation for three patients	c.1608+1G>A	Case 1: 80 days Case 2: 5 months Case 3: 48 days Case 4: 29 days	Case 1: 1 month Case 2: 5 years Case 3: 12 days Case 4: 4 days	Cases 1, 2, and 4: Not indicated Case 3: 30 mg/day	Cases 1, 2. and 3: Not detailed Case 4: drop in PTH	Not evaluated	Not evaluated	No
Gulcan-Kersin et al. (41)	1	Ho CASR mutation	c.1836 G>A p.Gly613Glu	1 day	18 months	1.5 mg/kg/d up to 8 mg/kg/d	Normalization of calcium and PTH	Urine calcium levels remained stable at the normal range	Not evaluated	No
Abdullayev et al. (42)	1	Ho CASR mutation	c.679 C>T p. Arg227Ter	10 days	18 months	1.5 mg/kg/d up to 11 mg/kg/d	Persistant hyperPTH	Not evaluated	Not evaluated	No
Leunbach et al. (43)	1	He *CASR* mutation	c.1745G>A p.Cys582Tyr	29 days	8 months	0.5 mg/kg/day	Normalization of calcium	Not evaluated	Not evaluated	No
Tuli et al. (15)	1	Not available	Not applicable	14 years	3 months	1.3–8.5 mg/kg/day	Normalization of calcium and phosphate; persistent elevated PTH levels	Not evaluated	Not evaluated	No
Aubert-Mucca et al. (44)	1	He *CASR* mutation	c.554G>A p.Arg185Gln	22 days	11 months	0.5–3 mg/kg/day	Normalization of calcium and PTH	Nephrocalcinosis at 6 months	Normalization of bone abnormalities	No
Sunuwar et al. (45)	1	VDR Type II	Not applicable	2.5 years	12 months	0.25–0.5 mg/kg/day	Normalization of calcium, phosphate and PTH	Not evaluated	Resolution of wrist swelling	No

CaSR, Calcium Sensing receptor; He, Heterozygous; Ho, Homozygous; VDR, Vitamin-D Dependent Rickets.

A recent meta-analysis has evaluated 28 studies including 722 adults receiving cinacalcet for PHPT (because of contra-indication to surgical procedure or parathyroidectomy failure) (13). It reported a normalization of calcium levels in 90% of cases; PTH levels were significantly reduced but only 10% of cases normalized PTH levels (13). The effect of cinacalcet was greater when baseline calcium values were above 3 mmol/L. In our study, we observed a significant decrease of calcium levels after 1 month of therapy that was sustained until the last follow-up; no hypocalcemia was reported. All patients displayed calcium levels above 2.80 mmol/L at the time of cinacalcet initiation; twelve patients normalized their calcium levels after cinacalcet therapy with doses that remained stable.

In the present study, different PTH assays were used in the different centers, with different normal ranges, explaining why we presented PTH data as ULN-PTH. The ULN-PTH significantly decreased after 12 months under cinacalcet treatment, and we observed a nearly 50% decrease of PTH levels between cinacalcet initiation and the last follow-up. The patients nevertheless remained within the “normal” range during follow-up, even though the normal range in case of hypercalcemia remains quite challenging to define. The main objective for physicians was to obtain acceptable calcium levels allowing to obtain optimal growth with adequate nutritional calcium intake for age.

Hypercalcemia due to neonatal severe HPT (NSHPT) secondary to homozygous or heterozygous mutations of *CASR*, has been treated since 2011 with cinacalcet, in association with biphosphonates, in order to avoid post-surgical hypoparathyroidism (2, 16, 23, 25, 36, 40). Clinical presentation or response to treatment, such as Cinacalcet, may differ depending to *CASR* mutation (homozygous or heterozygous) and interindividual differences (46, 47). Some mutations only partially affect the CaSR function secondary to an alteration of the configuration of the calcium binding domain or a partial loss of the transduction domain. As suggested by Gulcan-Kersin et al. a therapeutic test with Cinacalcet could be started before genetic result to treat NSHPT or severe acute hypercalcemia and suspended if we do not observe hypercalcemia correction (41).

Maximum doses of cinacalcet were similar between the group with “*CASR* mutation” and “other etiologies” (1.0 mg/kg/day). The European consensus in pediatric dialysis recommends ≤ 0.2 mg/kg per day for the starting dose and 2 mg/kg per day for the maximal dose of cinacalcet (14). However, this consensus was established to stay on the safe side because of the risk of hypocalcemia in dialysis; here, in patients with hypercalcemia due to PHPT and normal renal function at baseline, the risk of hypocalcemia is lower.

In a meta-analysis of studies in adults, 23% of 722 patients suffered from nausea or vomiting and 3% had hypocalcemia (most of them are mild or asymptomatic); the majority of these adverse reactions were not severe enough to stop the treatment (13). In pediatrics (Table 4), one paper described gastrointestinal symptoms (29). Other reported adverse effects included mild headache and occasional numbness and tingling in legs but were not observed in our cohort (21, 28). Despite these favorable outcomes, we believe that it is of utmost importance to provide a global information to children and their caregivers on the risk and symptoms of hypocalcemia, on the risk of drug interactions and further QTc interval prolongation and not only through hypocalcemia with cinacalcet treatment. Indeed, a special caution should be given in children to the association between cinacalcet and macrolides or ondansetron, that is contra-indicated (48).

In the kidney, CaSR is expressed in all nephron segments; it plays a crucial role in calcium excretion (49, 50). Activating *CASR* mutations lead to hypercalcemia that reduces calcium and sodium transport in the Henle loop (Na-K-2Cl and paracellular pathways) through claudins, in association with a decreased urinary concentrating ability (51, 52). Our results are similar to previous reports, demonstrating stable renal function and no direct effects of cinacalcet on urinary calcium (22–24, 27, 28, 30, 35, 37). Only one paper has reported the onset of nephrocalcinosis after 6 months of cinacalcet therapy in a patient with heterozygous *CASR* mutation (44). Multiple *CASR* mutations have been identified (53) which reduce receptor function or produce truncated inactive CaSR and heterogenous response to cinacalcet have been observed and influence urinary calcium excretion (22–24, 27, 28, 30, 35, 37). *In vitro* studies showed that calcimimetics were able to restore sensitivity on intra- and extracellular *CASR* mutations and deactivations mutations (54, 55). This mechanism in the kidney is still under investigation and requires further studies.

This study has some limitations, and notably the (relatively) small number of patients. However, we are in the field of orphan diseases in pediatrics, with an off-label use of novel therapies; here, we almost double the number of reported pediatric patients having received cinacalcet as an off-label drug for PHPT. Data on bone metabolism (such as CTX, bone density) and calcium intake are unfortunately not available after cinacalcet treatment in the present study; however in most studies, growth improved or remained stable at the end of the follow-up, similarly to what is observed here (16, 23, 24, 27).

In conclusion, our observational data suggest that cinacalcet could be a safe alternative therapy in pediatric PHPT to treat hypercalcemia without major side effects. Even though European guidelines already propose this management as a second-line off-label therapy in adults with PHPT, larger international studies may evaluate its effect with the main objective to treat hypercalcemia and obtain optimal growth with normal calcium intake and vitamin D supplementation. Its long-term consequences especially for the potential risk of hypercalciuria and nephrocalcinosis later in life of these children should be further assessed.

## Data availability statement

The raw data supporting the conclusions of this article will be made available by the authors, without undue reservation.

## Ethics statement

The studies involving human participants were reviewed and approved by Comité d'Ethique des Hospices Civils de Lyon, session 18/02/2019, approval 19-26), and declared to the Information Technology and Liberty Commission (CNIL n°19-168). Written informed consent to participate in this study was provided by the participants' legal guardian/next of kin.

## Author contributions

JBe and JBa: data collection and writing the manuscript. SF: data collection. J-PS, CA, MC, ALie, LM, and ALin: physicians of patients from different center and proofreading of the manuscript. ID: statistical analysis required for the review. All authors contributed to the article and approved the submitted version.

## Conflict of interest

JBa is a clinical investigator for industry-sponsored clinical trials on the use of calcimimetics (cinacalcet and etelcalcetide) in pediatric dialysis (Amgen). JBa has received research grants from Amgen (RENOCLASTE study: ID-RCB 2017-A03241-52). The remaining authors declare that the research was conducted in the absence of any commercial or financial relationships that could be construed as a potential conflict of interest.

## Publisher's note

All claims expressed in this article are solely those of the authors and do not necessarily represent those of their affiliated organizations, or those of the publisher, the editors and the reviewers. Any product that may be evaluated in this article, or claim that may be made by its manufacturer, is not guaranteed or endorsed by the publisher.
